# Durability and Strength Characteristics of Casein-Cemented Sand with Slag

**DOI:** 10.3390/ma13143182

**Published:** 2020-07-16

**Authors:** Sung-Sik Park, Seung-Wook Woo, Sueng-Won Jeong, Dong-Eun Lee

**Affiliations:** 1 Department of Civil Engineering, Kyungpook National University, 80 Daehakro, Bukgu, Daegu 41566, Korea; sungpark@knu.ac.kr (S.-S.P.); geowsw@knu.ac.kr (S.-W.W.); 2 Korea Institute of Geoscience and Mineral Resources, Daejeon 34132, Korea; swjeong@kigam.re.kr; 3 Department of Architectural Engineering, Kyungpook National University, 80 Daehakro, Bukgu, Daegu 41566, Korea

**Keywords:** durability, strength, casein, soil binder, blast furnace slag

## Abstract

Casein is often used as an eco-friendly wood adhesive. In this study, we used casein for soil cementation by mixing it with Jumunjin sand, sodium hydroxide (SH), and calcium hydroxide (CH) as a standard casein formula. The modified casein binder with different proportions of SH and CH was applied to improve water resistance. Furthermore, a blast furnace slag (BFS) was additionally mixed and reacted with alkalinity of modified casein binder. Thus, three types (standard, modified, and modified + BFS, referred to as STD, MOD, and MBS, hereafter) of casein binders were tested for durability and strength of casein-cemented sand. A piezoelectric sensor was installed within each sample to determine the curing time of the casein-cemented samples. The samples were air-cured at room temperature for seven days and some were repeatedly immersed in water thrice. Unconfined compression and jar slake tests were carried out to evaluate the strength and durability of the casein-cemented sand. Also, the microstructure was analyzed using a scanning electron microscope (SEM). We observed variations of peak conductance and corresponding frequency converged as the curing time increased. It was most significant for the MBS samples, which developed strength early. The unconfined compressive strength (UCS) of the air-cured samples was higher than those repeatedly immersed in water due to wash-off of the casein binder. The UCS of the dry MBS sample was 9900 kPa while that of the immersed sample was 430 kPa, which gradually decreased to 60 kPa upon repeated immersion. The samples with STD and MOD had no resistance to durability and showed cracks on the surface, while the MBS sample exhibited significantly improved durability and no cracks. We found that the MBS binder had a positively significant effect on the durability and strength of casein-cemented sand.

## 1. Introduction

In general, soil cementation, using various binders, is one of the ground improvement techniques to control or enhance the strength, durability, permeability, and other engineering properties of soil [1]. Cement is the most common binding agent for cementation that induces hardening behavior for various types of soil [2]. Besides, cement is cheaper than other binding agents for construction [3]. Despite having many benefits of improving the engineering properties of soil, cement causes environmental problems such as CO_2_ emission and natural environmental damage. Therefore, several new soil binders (including biopolymer, gum, and resin) have been developed to improve soil strength and reduce environmental problems [4,5,6,7,8].

Chang et al. [9] investigated soil cementation using casein protein from waste bovine milk. Casein is a predominant protein in milk and can be obtained from milk treatment [10]. When casein is mixed with an alkaline substance, it exhibits an adhesive property [11]. Some casein adhesives have been used in wood furniture and for glass bottle labels due to the adhesive’s excellent efficiency at low temperatures [12]. On the other hand, blast furnace slag (BFS) is a residual material produced from the rapid water quenching of a furnace. In general, BFS has a relatively high chemical reactivity due to its unstable crystal structure [13]. It undergoes hydration slowly with slag and water. However, when in contact with an alkaline solution, cementation occurs at once [14]. A lot of chemical agents that activate the curing reaction of slag have been reported [15,16,17]. A casein binder shows weak durability and low strength with water. Having a reaction of hydration in the alkaline condition, BFS, made of some alkaline ingredients, can be added to overcome such a weakness of a casein adhesive.

Because cemented soils usually have lower strength than concretes or rocks, the evaluation of the durability of the cemented soils is hindered. Therefore, a jar slake test can be used to evaluate the durability of weakly cemented soils. Also, for low-strength materials, it is important to predict the curing time for field applications. Lee et al. [18] used piezoelectric materials (PZT, i.e., lead zirconate titanate) to analyze the setting of cement mortar. Elsewhere, Park et al. [19] applied PZT to a predicting unit weight of sand. Such piezoelectric elements can be used to evaluate the physical and mechanical properties of cementitious materials.

In this study, three types of casein binders (standard, modified, and modified + BFS, referred to as STD, MOD, and MBS, hereafter) were prepared to enhance the durability and strength of sand samples at different (dry or wet) curing condition for seven days. Some samples were repeatedly immersed thrice in water. A PZT sensor was embedded in the Jumunjin sand to measure the conductance and frequency of the PZT during curing. The curing time and corresponding strength were investigated based on the peak conductance and corresponding frequency. The unconfined compression and durability tests were conducted to evaluate the casein binder in terms of strength and water resistance. The strength loss from repeated wetting was also estimated on each water immersion. The microstructure of the samples was examined through scanning electron microscope (SEM) analysis for each binder.

## 2. Materials and Testing Methods

### 2.1. Materials

#### 2.1.1. Jumunjin Sand and Blast Furnace Slag

**Figure 1:**
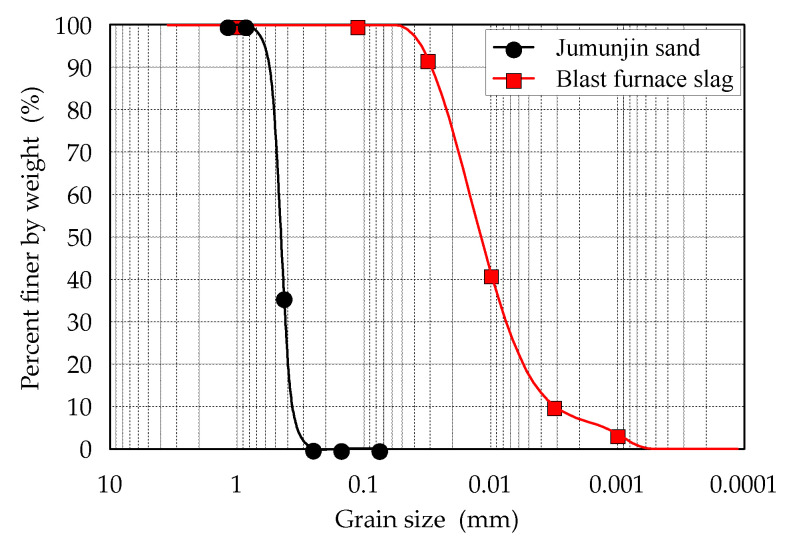
Grain size distribution curves of Jumunjin sand and blast furnace slag (BFS).

**Figure 2:**
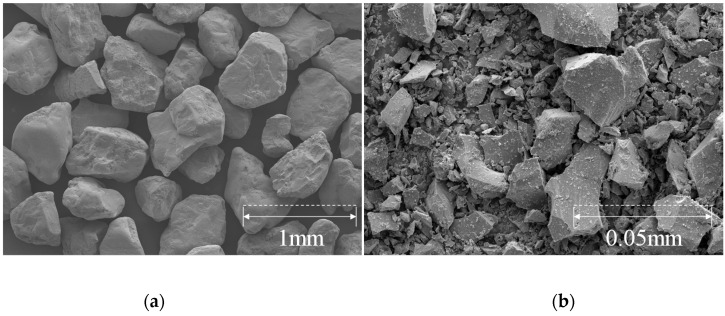
Particle shape of the used materials. (**a**) Particle shape of Jumunjin sand, (**b**) Particle shape of blast furnace slag.

Jumunjin sand was used for cementing a soil with casein adhesive. The sand is a standard in Korea, classified as a poorly graded sand (SP) in the Unified Soil Classification System (USCS). The sand contains >87% of silica (SiO_2_) and 64% of the particles are in the size range of 0.425–0.85 mm. The uniformity coefficient (Cu) and coefficient of gradation (Cc) of the soil are 1.61 and 1.03, respectively. We also obtained blast furnace slag (BFS) which was composed of 52.62% of CaO, 34.32% of SiO_2_, and 15.29% of Al_2_O_3_. The composition of the BFS was similar to that of Monshi (1999) [20]. The particle size distribution curve and material properties of Jumunjin sand and BFS are shown in Figure 1 and listed in Table 1. Figure 2 shows particle images of Jumunjin sand and BFS. Furthermore, X-ray fluorescence (XRF) analysis was performed to obtain the chemical composition of Jumunjin sand and BFS. The results are summarized in Table 2 and Table 3, respectively.

#### 2.1.2. Casein Binders

The three types of binder (STD, MOD, and MBS) were prepared with different ingredient proportions, listed in Table 4. Because pure casein is poorly soluble in water, it requires the addition of an alkaline solution to solubilize. When basified, several ions in the aqueous solution evinced the binding properties of casein. The performance of the casein binder changes depending on the presence (type and magnitude) of cations and the OH^−^ ions. In this study, the ingredient composition proposed by the Wisconsin Wood Research Institute was adopted as standard [21]. On the other hand, Lay et al. [22] mentioned that water resistance would increase when the calcium hydroxide is 30% composition. Although the standard binder was presented by numerous case studies, the modified casein binder was proposed to improve the water resistance of the standard casein binder. Furthermore, to enhance the water resistance, the BFS was added to the modified casein binder in 0.5×, 1.0×, and 1.5× of other constituents in the materials, except distilled water. Therefore, the ratio of blast furnace slag to casein (SCR) was 0.5, 1.0, and 1.5.

#### 2.1.3. Sample Preparation

Three types of casein-cemented sand were tested and noted as STD, MOD, and MBS in this study. The binder content and blast furnace slag–casein ratio (SCR) are listed in Table 5. The diameter and height of the cemented sample are 5 and 10 cm for the unconfined compression, water content, and piezoelectricity response tests. The height of the samples for jar slake test was 3 cm. A cubic sample with a 1 cm side length was used for the SEM analysis. The dry unit weight of all cemented samples was designed between 15–16 kN/m^3^. The samples were cured in the air and water at 20 °C. A piezoelectric element was installed at the center of each sample to examine the solidity process of the samples at different curing periods. All samples were kept in the mold for 24 h, then they were demolded and cured before further testing.

### 2.2. Testing Methods

#### 2.2.1. Lead Zirconate Titanate (PZT) Sensor

**Figure 3:**
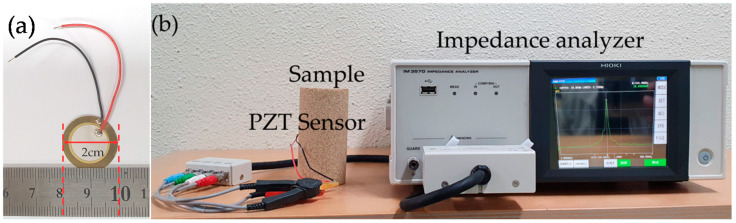
Images of (**a**) the lead zirconate titanate (PZT) sensor and (**b**) the measuring system with the PZT-embedded sample connected to an impedance analyzer.

The PZT sensor is 0.3 mm thick and 20 mm in diameter (Figure 3a). The sensor, connected to an impedance analyzer, was installed in the middle of the STD, MOD, and MBS samples strengthened with casein (Figure 3b). The binder content of the samples used was 4% and the SCR of the MBS binder was 1.0. The impedance analyzer collected the PZT sensor measurements (Hioki, Nagano, Japan). These measurements can reveal changes in the strength of the material at an early stage. The conductance-frequency response was recorded to analyze the solidity of the sand samples at the initial state, and at the curing ages at 12 h intervals for 7 days. The measured frequencies from the electrical impedance of PZT were in the range of 100–300 kHz. The water content of the samples was measured after the 3rd day of curing and was used as an additional parameter of sample solidity analysis.

#### 2.2.2. Scanning Electron Microscope (SEM) Analysis

The SEM image analysis (Hitachi, Tokyo, Japan) was carried out to examine the microstructure and the interactions between casein and sand samples. The cemented sand was visualized by magnifying the image successively at 100× and 400× to visualize the bonds among the casein, slag, and sand particles. A 4% binder content was used for each sample and SCR of the MBS binder was 1.0.

#### 2.2.3. Unconfined Compression Test

**Figure 4:**
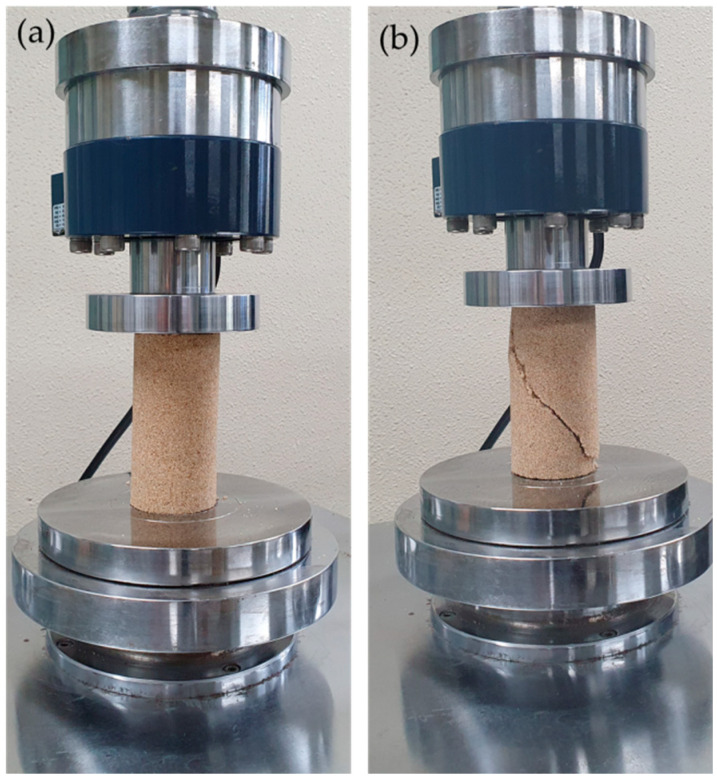
(**a**) Sample under unconfined compression and (**b**) sample failure mode.

The unconfined compression test on casein-cemented sand was performed according to the American society for Testing and Materials (ASTM) D 2166 [23]. The load cell, with a capacity of 5 t, was attached to the actuator to perform the test at dry and wet conditions (Figure 4). The load was applied at a rate of 1.0 mm/min. The wet condition was achieved by immersing the samples in water before the experiment. For each sample type, an average of three measurements was obtained for UCS and axial strain. The elastic modulus was computed as the tangent slope of half the stress point from the compressive stress-strain curves.

#### 2.2.4. Jar Slake Test

The casein binder resists water. However, when in contact with water for a long time, the binding structure becomes weak progressively. Thus, the STD, MOD and MBS samples cured for 7 days were immersed in water and the degree of degradation from the Jar slake test was measured. All samples were cemented with 4% binder content and SCR of MBS was 1.0. Details of the jar slake test have been documented [24,25,26]. This method expresses the degree of degradation of a sample in water over time. The samples were classified into Categories 1–6, depending on the degree of decomposition that is recorded every 30 min for about 24 h. Samples in Category 1 degraded to a uniform mud-like structure, Category 2 reduced completely to flakes, Category 3 broke slowly and develop few chips, Category 4 broke rapidly with many fractures, Category 5 broke slowly and with many fractures, and Category 6 showed no observable change.

## 3. Results and Discussions

### 3.1. Results of Lead Zirconate Titanate (PZT) Sensor Analysis

**Figure 5:**
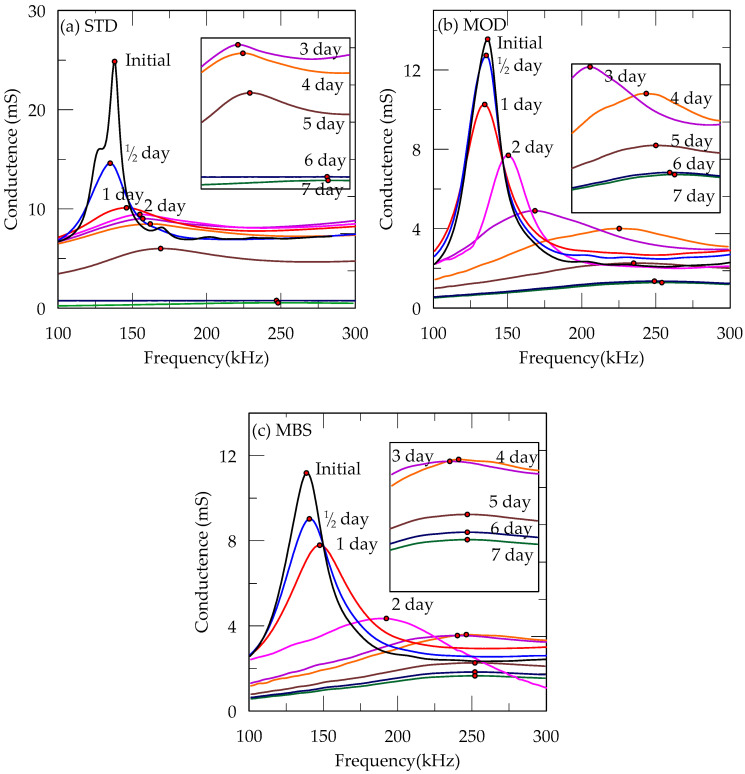
Conductance spectral responses from the PZT sensor placed in (**a**) standard STD, (**b**) modified (MOD), and (**c**) modified + BFS (MBS) binder-strengthened sand.

Figure 5 shows the conductance spectral response of the embedded PZT sensor of STD, MOD, and MBS samples with curing time. The initial state of the sample when placing the mixture in the mold was defined as a reference point. The peak conductance occurred due to resonance by alternating current flow. It was shown that a peak point of conductance disappeared and flattened with the curing period. Such flat-shaped responding spectra were observed from all three binder samples with the curing time. The disappearance of peak means that the confining condition around the sensor has been hardened. Thus, we noticed that the internal solidity progress of the sample sufficiently occurred around the PZT sensor.

**Figure 6:**
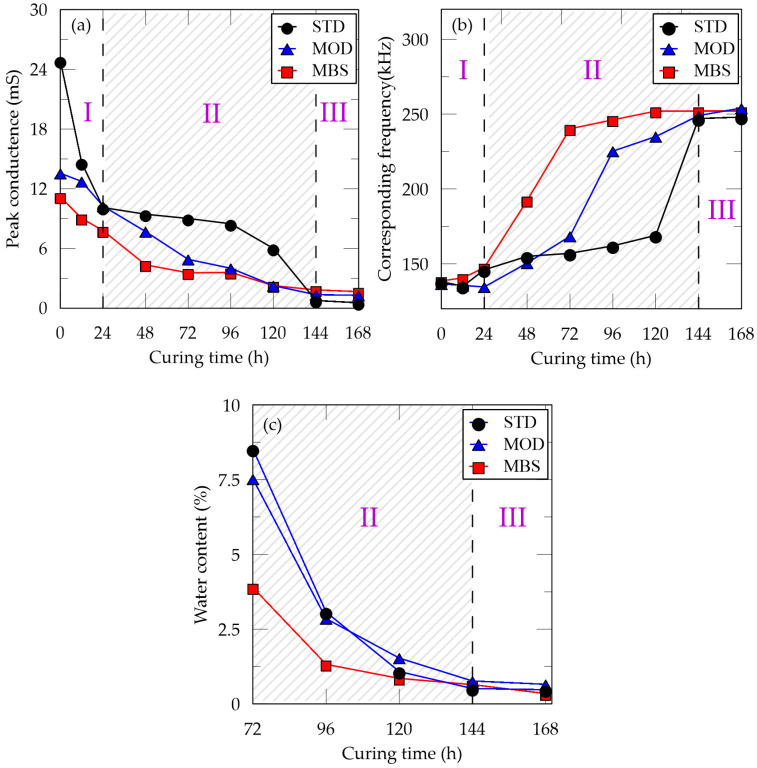
Variations of (**a**) peak conductance, (**b**) corresponding frequency, and (**c**) water content over 7 days.

In Figure 6, the variations of peak conductance, corresponding frequencies, and water content over a curing period of seven days are shown. In Table 6, the peak conductance at the initial state of STD, MOD, and MBS samples were 24.86, 13.55, and 11.19 mS, respectively, with corresponding frequencies of 138, 136, and 138 kHz. As the samples cured for 1/2, 1, 2, 3, 4, 5, 6, and 7 days, their peak conductance gradually decreased and converged approximately at 0.53, 1.28, and 1.65 mS, respectively. On the other hand, their corresponding frequencies gradually increased and converged approximately at 248, 254, and 253 kHz, respectively.

From Figure 6a,b, the variation of conductance and frequency can be separated into 3 stages: (I) 0–24 h, (II) 24–144 h, and (III) 144–168 h. From our observation, the cementation process was of three stages: exterior cementation, interior cementation, and sand-binder stabilization. At the first stage, peak conductance decreased but the corresponding frequency changed slightly, and the interior sand surrounding the sensor was soft, whereas the exterior sand was hard. Although the vibration of freedom and resonant frequency were maintained due to the soft surroundings, the peak conductance decreased gradually. However, at the second stage, the cementation of the interior sand occurred, and both the peak conductance and corresponding frequency moved significantly. Finally, the motions started to converge like that of the III stage. The time difference between the external hardening and internal hardening was due to the extent of moisture reduction. Because the moisture of the sample evaporated from the exterior to the interior, the water content decreased gradually till the 5th day, becoming lower than 1% in the 6th and 7th days.

The variation of the water content was separated with the same time section with other results as shown in Figure 6c. During curing, the water content of MBS samples was lower than those of STD and MOD samples and converged earlier. We observed that both the water evaporation and hydration of the blast furnace slag occurred. Therefore, the 7-day period was enough for curing and adding BFS in the binder contributed to achieving a rapid hardness and sufficient strength development.

### 3.2. Results of Scanning Electron Microscope (SEM) Analysis

**Figure 7:**
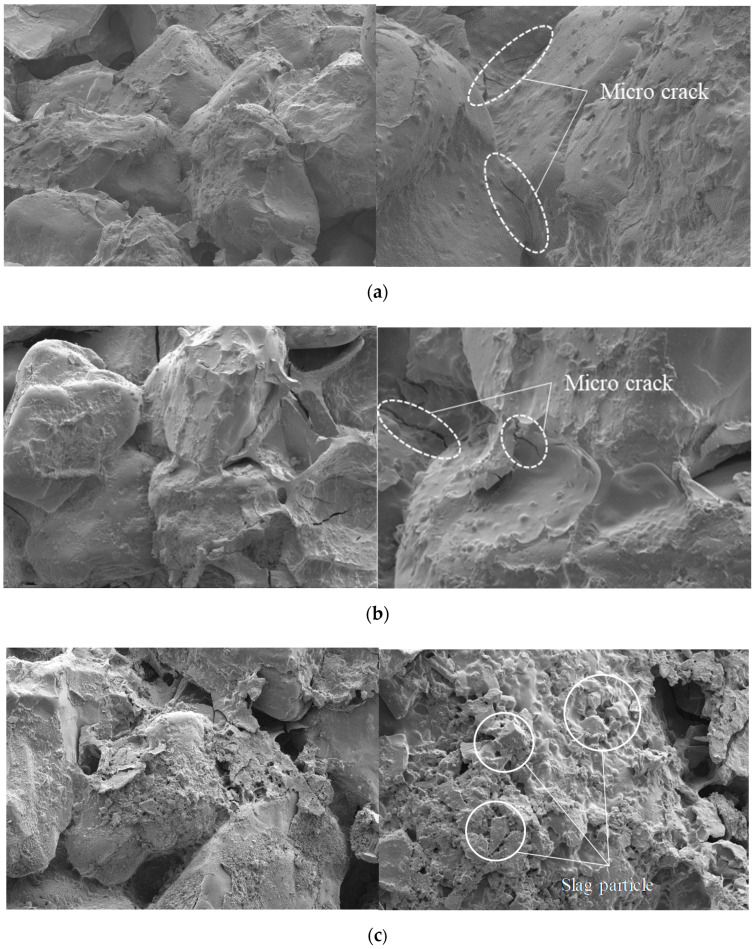
Results of SEM image analysis (Left: x100, Right: x400). (**a**) Scanning electron microscope (SEM) images of STD samples, (**b**) SEM images of MOD samples, (**c**) SEM images of MBS samples.

The SEM analysis was performed to visualize the microstructure and bonds between particles of casein, slag, and sand samples as shown in Figure 7. We observed that casein enveloped the sand and enhanced the bond between sand particles. Figure 7a–c present the interaction of particles for STD, MOD, and MBS samples, respectively. The casein bonded the sand particles effectively without voids on the contact surface. The micrographs of STD and MOD showed some microcracks on the surface of the casein bond, attributed to water evaporation. The volume of the binder bond reduced during moisture evaporation of the casein binder, causing the inner crack of the structure. However, the MBS sample did not exhibit any crack on the binder structure, probably due to the presence of both the blast furnace slag and casein bonders in the mixture. Unlike STD and MOD samples, the slag contained in the MBS samples reacted with moisture and removed the micro-cracks caused by moisture evaporation.

### 3.3. Results of Unconfined Compression Test

**Figure 8:**
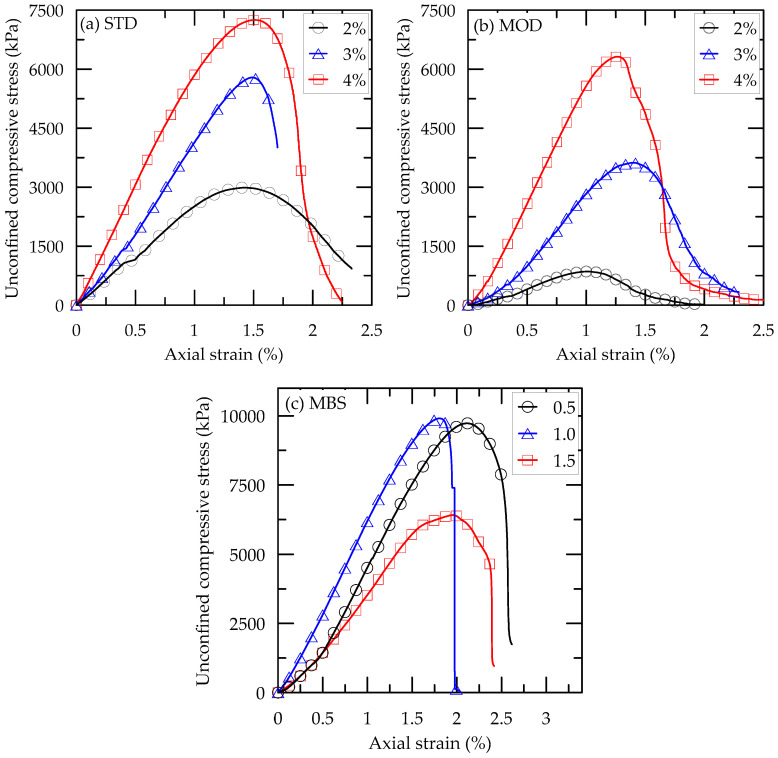
Stress-Strain relationship of casein and/or slag cemented (**a**) STD, (**b**) MOD, and (**c**) MBS samples.

The relationship between unconfined compressive stress and axial strain of the casein-cemented samples (STD, MOD, and MBS) is shown in Figure 8, while the results are summarized in Table 7, with those of ordinary Portland cement (OPC) [27]. The maximum UCS values were 7249, 6313, and 9908 kPa for STD, MOD, and MBS, respectively. Each of STD, MOD, and MBS samples showed a UCS value higher than that of OPC. The UCS of STD samples was significantly higher than that of MOD samples, irrespective of the binder content. The adjustment of calcium hydroxide and sodium hydroxide ratio in the STD binder lowered the strength of MOD samples. On the other hand, the addition of slag into MOD samples caused a significant increase in the UCS with SCR of 0.5 and 1.0. When a SCR increased to 1.5, the increase in the UCS of the MBS sample was not noticeable because the slag supplied was in excess, therefore, it could not react with water. Moreover, the slag was not hydrated inside the sample, which resulted in strength reduction. Therefore, the slag and casein ratio of 1:1 was a suitable mixing ratio for developing high-strength cement.

**Figure 9:**
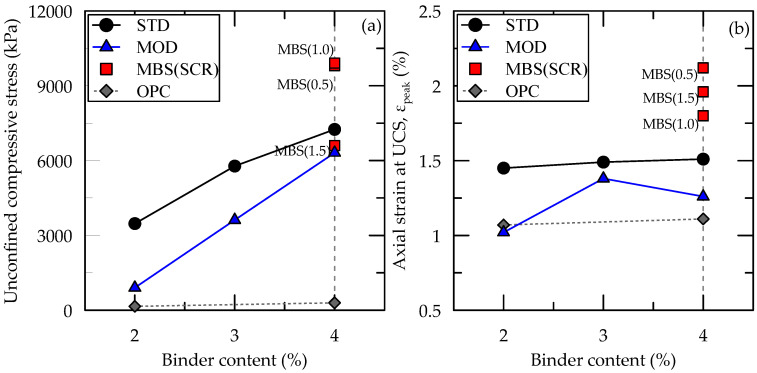

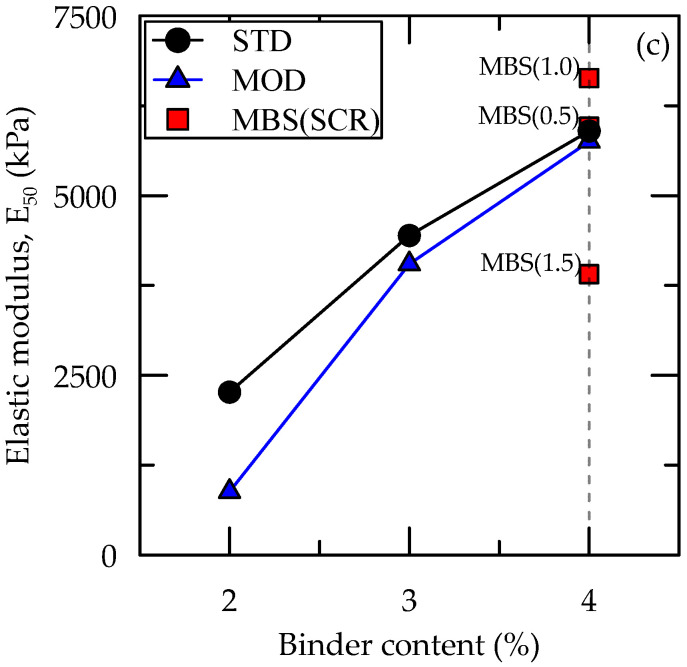
(**a**) The UCS, (**b**) axial strain at UCS, (**c**) E_50_ of the cemented samples.

Furthermore, the dry unit weights were similar to the designed values. The weights increased slightly and proportionately with the binder content and SCR. The axial strains at the maximum stress (ε_peak_) of all samples ranged from 1% to 2% (Figure 8). These values, compared in Figure 9b, are generally higher than that cemented with OPC. As shown in Figure 9b, the ε_peak_ of STD samples slightly increased as the binder content increased. Generally, the ε_peak_ of MOD and MBS samples showed no distinct pattern with increasing binder content. This was because the maximum binder content was relatively small and the developed strength was also low. Similar behavior was also observed with the OPC-cemented sand. On the other hand, the ε_peak_, usually, decreased with an increased binder content as a concrete, cement mortar, and cement paste. These materials have much higher strength and brittle behavior compared to cemented sands.

Generally, the elastic modulus (E_50_) was between 2 and 6 MPa as shown in Figure 9 and compiled in Table 7. The STD, MOD, and MBS samples showed higher E_50_ to that of OPC. The value increased with the binder content. The maximum UCS was obtained at lower strain, leading to the increased elastic modulus. However, this behavior was not clearly shown in this study, in which the ε_peak_ was an insignificant difference. A similar result was obtained in a previous study as a result of mobilizing the shearing resistance at the interaction between sand particles after breaking of cemented bonds [28]. Therefore, ε_peak_ does not decrease with an increase in UCS and E_50_, because the friction among the sand particle sustains the shearing resistance, losing the resistance of the cementing bonds.

### 3.4. Results of Durability Test

#### 3.4.1. Jar Slake Test

Jar slake test was carried out to assess the durability of casein-cemented sand. The results were summarized in Table 8 as Category 1 to 6 (C1–C6). For the STD samples, degradation or separation of particles started after 1 h of soaking. When the soaking time reached 3 h, the particles were completely decomposed and disintegrated (C1). This indicated that the cemented sand with standard casein binder was weak in water. Similarly, the MOD samples were completely disintegrated after 20 h, whereas, the MBS samples withstand the immersion in water for 24 h (C6). Therefore, the MBS binder evinced an excellent hydrophobic characteristic, without any cracks or separation within its structure.

#### 3.4.2. Repeated Soaking Test

Because the MBS samples did not disintegrate during the jar slake test, they were selected for further strength evaluation by repeated soaking in distilled water. The wetting and drying cycle was repeated up to 3 times. The curing conditions of all the samples are summarized in Table 9. The influence of water on the UCS was achieved by comparing the UCS after immersion to that of the air-dried samples. A decrease in UCS of the repeatedly wetted samples was described by Park (2010). Table 10 summarizes the results of the unconfined compression test of the repeatedly soaked samples.

**Figure 10:**
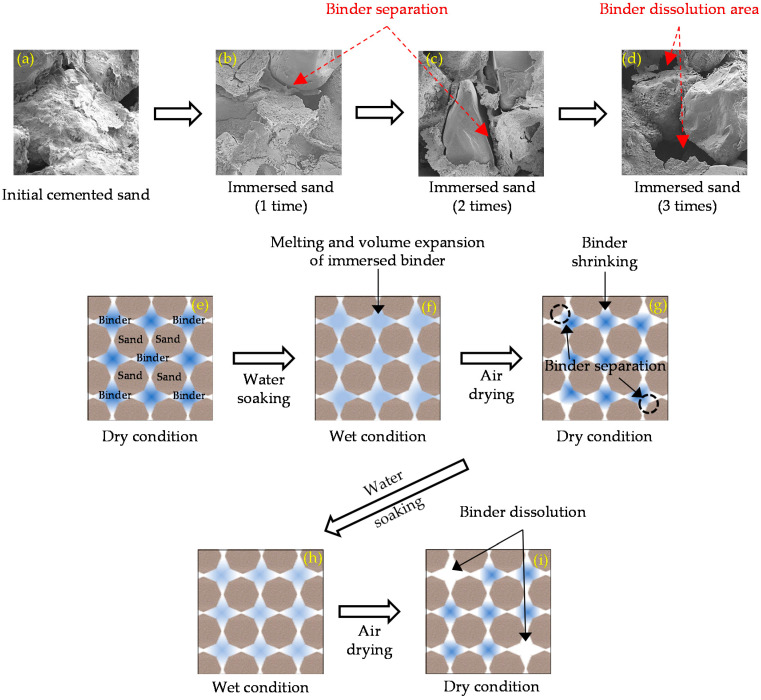
SEM micrographs of repeatedly soaked samples (**a**–**d**), and the diagrams of particle behavior (**e**–**i**).

Compared to the sample submerged once, the UCS decreased approximately 4 and 6 times after the 2nd and 3rd immersions, respectively. This weakening behavior is attributed to the progressive weakening of the bond between casein, slag, and sand particles. In Figure 10a through Figure 10d, the SEM micrographs of the air-dried samples and the soaked samples are depicted. As the immersion was repeated, the binder and sand particles were disjointed. Figure 10d illustrates the dissolution of the binder in water as it melted in the solution. Based on the difference in particle behavior at both dry and after repeated immersion, the process of dissolution of the binding materials is described in Figure 10e through Figure 10i. The moisture was absorbed by the connecting binder, causing its expansion and decomposition (Figure 10f). During drying, binder volume gradually decreased as the moisture evaporated (Figure 10g), with no evidence of binder separation. Consequently, the binder begins to dissolve at the third immersion (Figure 10h), resulting in the lowest compressive strength after the third soaking, due to the voids and weak bond between the casein, slag, and sand particles.

## 4. Conclusions

This study investigated the engineering properties of cemented sands with standard (STD), modified (MOD), modified + BFS (MBS) casein binders. The strength development was analyzed as well as the unconfined compressive strength (UCS) and durability of cemented sand with the binders. The following conclusions were drawn:

1. In the solidity performance test using a PZT sensor, there was a continuous decrease in the peak conductance in all samples as the curing time increased until the 6th day, after which it plateaued. Also, the water content was lower than 1% from the 5th day. The curing period of 7 days was enough to harden the casein-cemented sand.
2. The UCS and elastic modulus increased proportionately with the binder content for all STD, MOD, and MBS samples. The hydrate reaction of the blast furnace slag added in the binder appeared, and the UCS of the MBS sample (9806 kPa) was the highest, 1.35, 1.55, and 22 times higher than those of STD, MOD, and 6% ordinary Portland cement (OPC), respectively. Also, the UCS improved most when the same amount of casein binder and blast furnace slag was used.
3. MBS binder was the most effective in enhancing the hydrophobicity and durability of the cement. The samples with MBS resisted in water even for 24 h without any separation and surface cracks. On the other hand, the STD and MOD samples collapsed in water in 3 and 20 h respectively.
4. Although MBS samples showed great durability compared with other samples, their UCS decreased approximately 4 times, and 6 times whenever it was immersed twice, and 3 times, respectively. The bond between casein, slag, and sand particles becomes weaker with repeated submersion in water.
5. The sands cemented with the casein revealed higher UCS than those of OPC sand when they were cured for seven days. However, their UCS decreased as the time of contact with water increased, and it was lower than the UCS of OPC when it contacted with water over than 24 h. We suggest the use of casein binder where high strength is required for a short time.

## Figures and Tables

**Table 1:** Physical properties of Jumunjin sand and BFS.

Material	D_60_ (mm)	D_30_ (mm)	D_10_ (mm)	Coeff. of Uniformity, Cu	Coeff. of Gradation, Cc	Unified Soil Classification System
Jumunjin sand	0.50	0.40	0.31	1.61	1.03	Poorly graded sand (SP)
Blast furnace slag	0.015	0.007	0.003	5	1.09	-

**Table 2:** Chemical components of Jumunjin sand.

	SiO_2_	Al_2_O_3_	K_2_O	Na_2_O	Fe_2_O_3_	CaO	BaO	Cl	L.O.I
Component (%)	87.70	6.61	4.03	0.76	0.25	0.11	0.09	0.07	0.38

**Table 3:** Chemical components of BFS.

	CaO	SiO_2_	Al_2_O_3_	MgO	SO_3_	TiO_2_	K_2_O	Fe_2_O_3_	MnO	L.O.I
Component (%)	42.62	34.32	15.29	3.25	1.99	0.73	0.45	0.43	0.40	0.52

**Table 4:** Composition of three casein binders.

Binder Ingredient	Standard Casein (g)	Modified Casein (g)	Modified Casein + BFS (g)
Casein	100	100	100
Distilled water	250	250	250
Calcium hydroxide	20	26	26
Sodium hydroxide	11	5	5
Blast furnace slag	-	-	65.5, 131, 196.5

**Table 5:** Types of binder and binder content.

ID	Binder Type	Binder Content (%)	Blast Furnace Slag/Casein (SCR)
STD	Standard	2, 3, 4	0
MOD	Modified	2, 3, 4	0
MBS	Modified + BFS	4	0.5, 1.0, 1.5

**Table 6:** Result of the PZT signal measurement.

ID		Initial	½ Day	1 Day	2 Day	3 Day	4 Day	5 Day	6 Day	7 Day
STD	P. C. * (mS)	24.86	14.61	10.11	9.44	9.01	8.48	6.00	0.76	0.53
	C. F. ** (kHz)	138	135	146	155	157	162	169	247	248
	Water content (%)	-	-	-	-	8.52	3.07	1.08	0.51	0.47
MOD	P. C. (mS)	13.55	12.73	10.26	7.69	4.90	4.00	2.26	1.35	1.28
	C. F. (kHz)	137	136	135	151	168	225	235	249	254
	Water content (%)	-	-	-	-	7.52	2.85	1.53	0.763	0.65
MBS	P. C. (mS)	11.19	9.03	7.79	4.35	3.55	3.59	2.26	1.83	1.65
	C. F. (kHz)	139	141	148	192	240	246	252	252	252
	Water content (%)	-	-	-	-	3.89	1.32	0.85	0.64	0.34

* P. C.: Peak conductance; ** C. F.: Corresponding frequency.

**Table 7:** Summary of the unconfined compression test results after 7 days of curing in air.

ID	Binder Type	Curing Condition	SCR	Binder Content (%)	Dry Unit Weight, γ_d_ (kN/m^3^)	Unconfined Compressive Strength (kPa)	Axial Strain at UCS, ε_peak_ (%)	Elastic Modulus, E_50_ (kPa)
STD	Standard	7 days in air	0	2	15.51	3472	1.45	2265
				3	15.84	5778	1.49	4444
				4	15.92	7249	1.51	5898
MOD	Modified	7 days in air	0	2	15.49	902	1.02	882
				3	15.81	3616	1.38	4049
				4	15.89	6313	1.26	5752
MBS	Modified + BFS	7 days in air	0.5	4	16.05	9806	2.12	5959
			1		16.17	9908	1.80	6635
			1.5		16.30	6600	1.96	3906
OPC	Ordinary Portland cement	7 days in air	0	2	-	155	1.07	117
				4	-	296	1.11	322
				6	-	444	1.10	436

**Table 8:** Result of the jar slake test.

ID	Binder Type	Binder Content (%)	SCR	Jar Slake Category at Soaking Time							
				3 h	6 h	9 h	12 h	15 h	18 h	21 h	24 h
STD	Standard	4	-	C1	C1	C1	C1	C1	C1	C1	C1
MOD	Modified	4	-	C6	C6	C4	C3	C3	C3	C1	C1
MBS	Modified + BFS	4	1.0	C6	C6	C6	C6	C6	C6	C6	C6

**Table 9:** The curing condition of the repeated soaking test.

Binder Type	Case	1 Day	2 Day	3 Day	4 Day	5 Day	6 Day	7 Day
Modified + BFS	Wet-1	Mold	Water	UCS test				
	Wet-2	Mold	Water	Air	Water	UCS test		
	Wet-3	Mold	Water	Air	Water	Air	Water	UCS test

**Table 10:** Results of unconfined compression test of the repeatedly soaked samples.

ID	Binder Type	SCR	Binder Content (%)	Case	Unconfined Compressive Strength (kPa)	Axial Strain at UCS, ε_peak_ (%)	Elastic Modulus, E_50_ (kPa)
MBS	Modified + BFS	1.0	4	Wet-1	436	2.99	229
				Wet-2	120	4.06	39
				Wet-3	68	3.51	24

## Acronyms

SH	Sodium Hydroxide
CH	Calcium Hydroxide
BFS	Blast Furnace Slag
STD	Standard Casein Binder
MOD	Modified Casein Binder
MBS	Modified + Blast Furnace Slag Casein Binder
UCS	Unconfined Compressive Strength
PZT	Lead Zirconate Titanate
SEM	Scanning Electron Microscope
USCS	Unified Soil Classification System
Cu	Uniformity Coefficient
Cc	Coefficient of Gradation
XRF	X-Ray Fluorescence
SCR	Ratio of Blast Furnace Slag to Casein
P.C.	Peak Conductance
C.F.	Corresponding Frequency
OPC	Ordinary Portland Cement
ε_peak_	Axial Strains at the Maximum Stress
E50	Elastic modulus
C1-C6	Jar slake Test Category
